# Percutaneous debulking strategy for severe nodular calcification in common femoral artery

**DOI:** 10.1186/s42155-022-00301-6

**Published:** 2022-05-27

**Authors:** Tetsuya Nomura, Issei Ota, Satoshi Tasaka, Kenshi Ono, Yu Sakaue, Keisuke Shoji, Naotoshi Wada

**Affiliations:** Department of Cardiovascular Medicine, Kyoto Chubu Medical Center, 25, Yagi-Ueno, Yagi-cho, 629-0197 Nantan City, Kyoto, Japan

**Keywords:** Endovascular treatment, Common femoral artery, Nodular calcification, Debulking, Myocardial biopsy forceps

## Abstract

**Background:**

Despite marked progress in endovascular treatment (EVT) techniques and devices, calcified lesions remain one of the toughest obstacles to EVT success. Moreover, because the common femoral artery (CFA) is known as a “non-stenting zone,” endovascular strategies for this area are controversial.

**Case presentation:**

Here we describe the technical tips for a novel, less invasive, and effective debulking strategy for severe nodular calcification using an endovascular maneuver. This technique was demonstrated in a 73-year-old man with severe calcified stenosis of the CFA. To complete a stent-less strategy for CFA, we conducted aggressive debulking of the nodular calcification, established a bidirectional approach from the radial artery and the superficial femoral artery (SFA), and inserted a balloon-guiding catheter in the SFA. Under distal protection provided by this catheter, we crushed the nodular calcification 43 times using myocardial biopsy forceps. After achieving a volume reduction of nodular calcification through this maneuver, we completed the procedure by inflating a 6-mm drug-coated balloon catheter. Final angiography demonstrated a reduced filling defect of the contrast medium in the CFA and favorable blood flow as far as the ankle. The puncture site on the SFA was closed with a vascular suture assisted by balloon inflation inside the vessel, which allowed the patient to be ambulatory immediately after the procedure without requiring bed rest.

**Conclusions:**

Severely calcified lesions in the CFA are usually difficult to treat using an endovascular strategy, but our novel and less invasive method may become a promising technique for managing these lesions.

**Supplementary information:**

The online version contains supplementary material available at 10.1186/s42155-022-00301-6.

## Introduction

Although marked progress has been made in the field of endovascular treatment (EVT) for peripheral artery diseases, calcified lesions remain a critical issue to obtain optimal EVT results (Cunnane et al. 2015). Atherectomy devices have recently become adjunctively important to debulk plaque within vessels, especially heavily calcified plaques, and greater plaque volume reduction usually results in more efficient balloon angioplasty and/or stenting. Furthermore, lesions in the common femoral artery (CFA) are usually treated with endarterectomy by vascular surgeons. However, some drawbacks of common femoral endarterectomy (CFE), such as wound complications, have been reported (Nguyen et al. 2015).

## Case presentation

A 73-year-old man with silent myocardial ischemia, hypertension, and dyslipidemia presented with claudication of the right foot (Rutherford class 3) after walking for 100 m. He was taking drugs for comorbidities and dual antiplatelet therapy for former coronary interventions. The right and left ankle-brachial pressure index (ABI) was 0.52 and 0.88, respectively. Ultrasound of the lower-extremity arteries showed a severe acoustic shadow in the right CFA, while a pulsed Doppler assessment distal to the CFA revealed an impaired flow pattern. Guidelines suggest CFE as the first-line treatment for this lesion type (Aboyans et al. 2017), but the patient desired less invasive EVT instead. Therefore, we performed EVT for the CFA lesions with severe nodular calcifications.

**Fig. 1:**
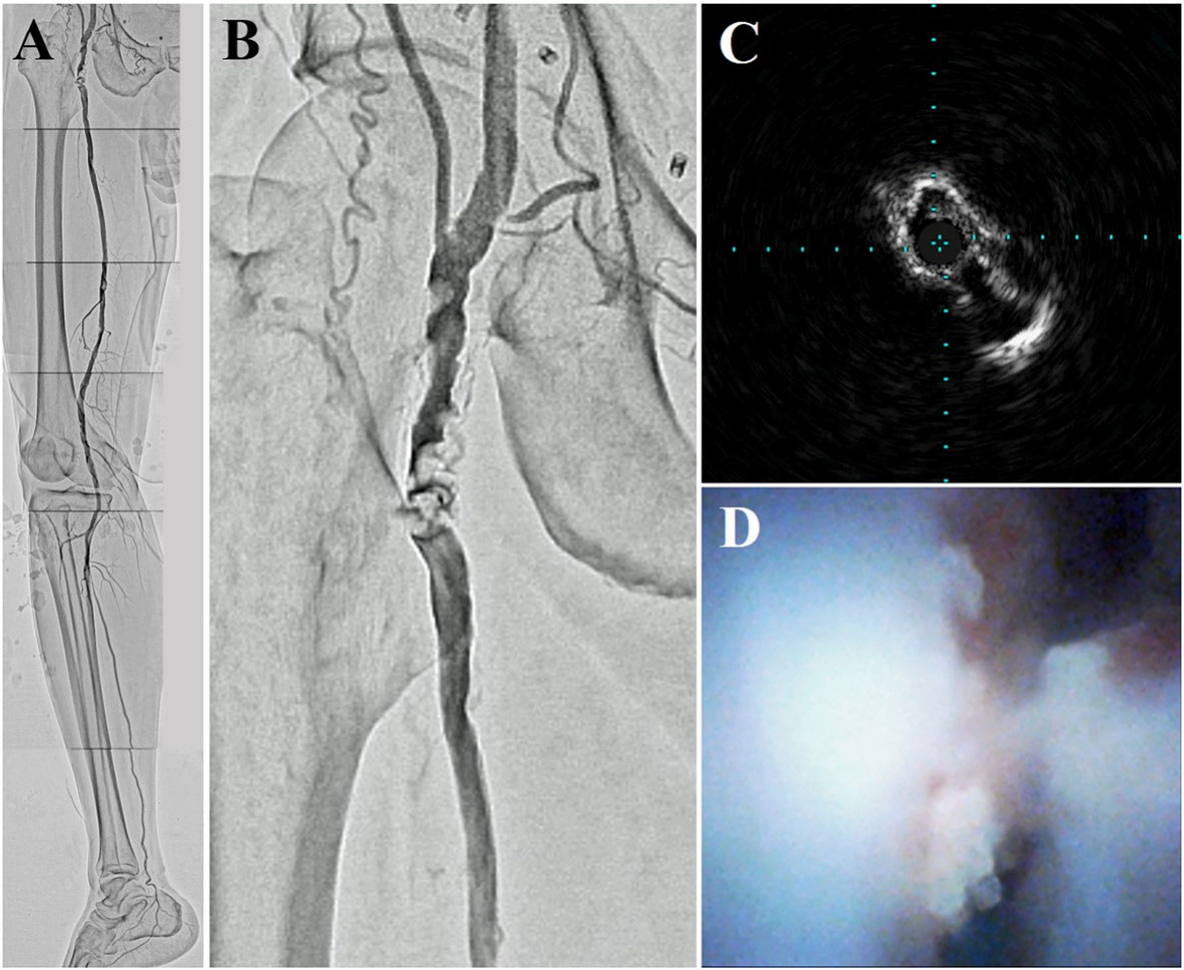
**A,B** Initial angiography showing a filling defect of contrast medium in the common femoral artery and one straight line through the posterior tibial artery. Initial intravascular ultrasonography (**C**) and angioscopy (**D**) images demonstrating a calcified mass protruding into the vascular lumen

**Fig. 2:**
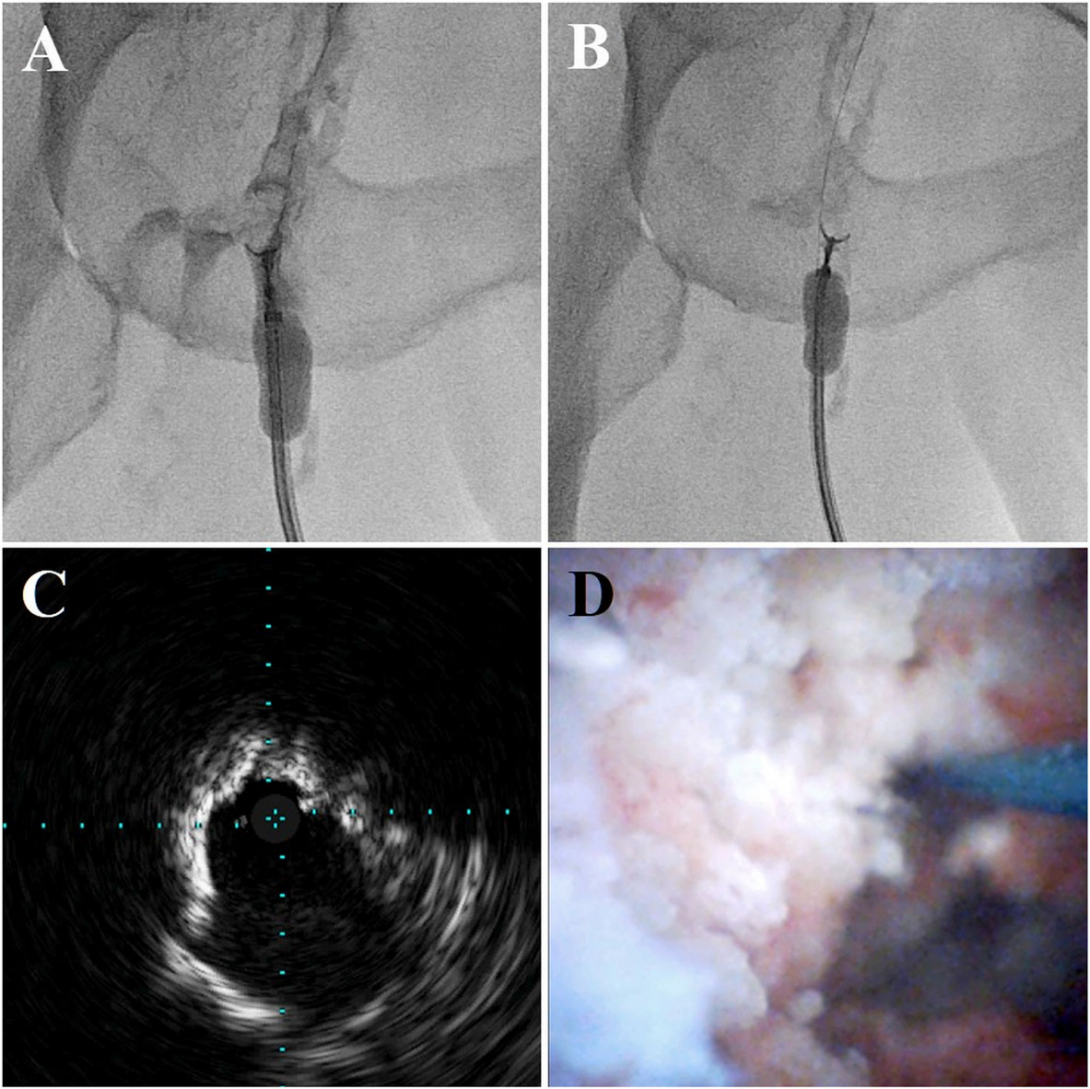
Debulking nodular calcification with forceps under distal protection with a balloon-guiding catheter (**A**, **B**; Videos 1 and 2). After multiple debulking procedures, intravascular ultrasonography (**C**) and angioscopy (**D**) showed luminal enlargement

We used the left radial artery (RA) because of heavy calcification at the puncture site in the contralateral CFA. A 7-Fr Glidesheath Slender (Terumo Corp., Tokyo, Japan) was inserted in the left RA. Immediately thereafter, an intra-arterial bolus of 5,000 units of unfractionated heparin was administered, and the activated clotting time was controlled to > 250 s. A 150-cm-long R2P SlenGuide guiding catheter (Terumo Corp., Tokyo, Japan), was placed just proximal to the left CFA. Initial angiography showed a clear filling defect of the contrast medium in the CFA (Fig. 1 A, B). Next, we punctured the proximal superficial femoral artery (SFA) under duplex ultrasound guidance and inserted a balloon-guiding catheter (OPTIMO PPI; Tokai Medical Products, Inc., Aichi, Japan) to enable ipsilateral retrograde access. After retrogradely advancing the floppy guidewire and avoiding the nodular calcification, we performed intravascular ultrasonography (IVUS) and angioscopy, which showed a mass of calcification protruding into the vascular lumen (Fig. 1 C, D). We crunched crushed the nodular calcification 43 times using myocardial biopsy forceps under distal protection provided by the OPTIMO PPI (Fig. 2 A, B; Videos 1, 2). We fully aspirated the debris trapped by the OPTIMO balloon through the catheter every 10 crushes. The calcified mass was notably shrunk on fluoroscopy, and IVUS showed favorable luminal enlargement (Fig. 2 C). Nodular calcification volume reduction was observed on angioscopy (Fig. 2D).

**Fig. 3:**
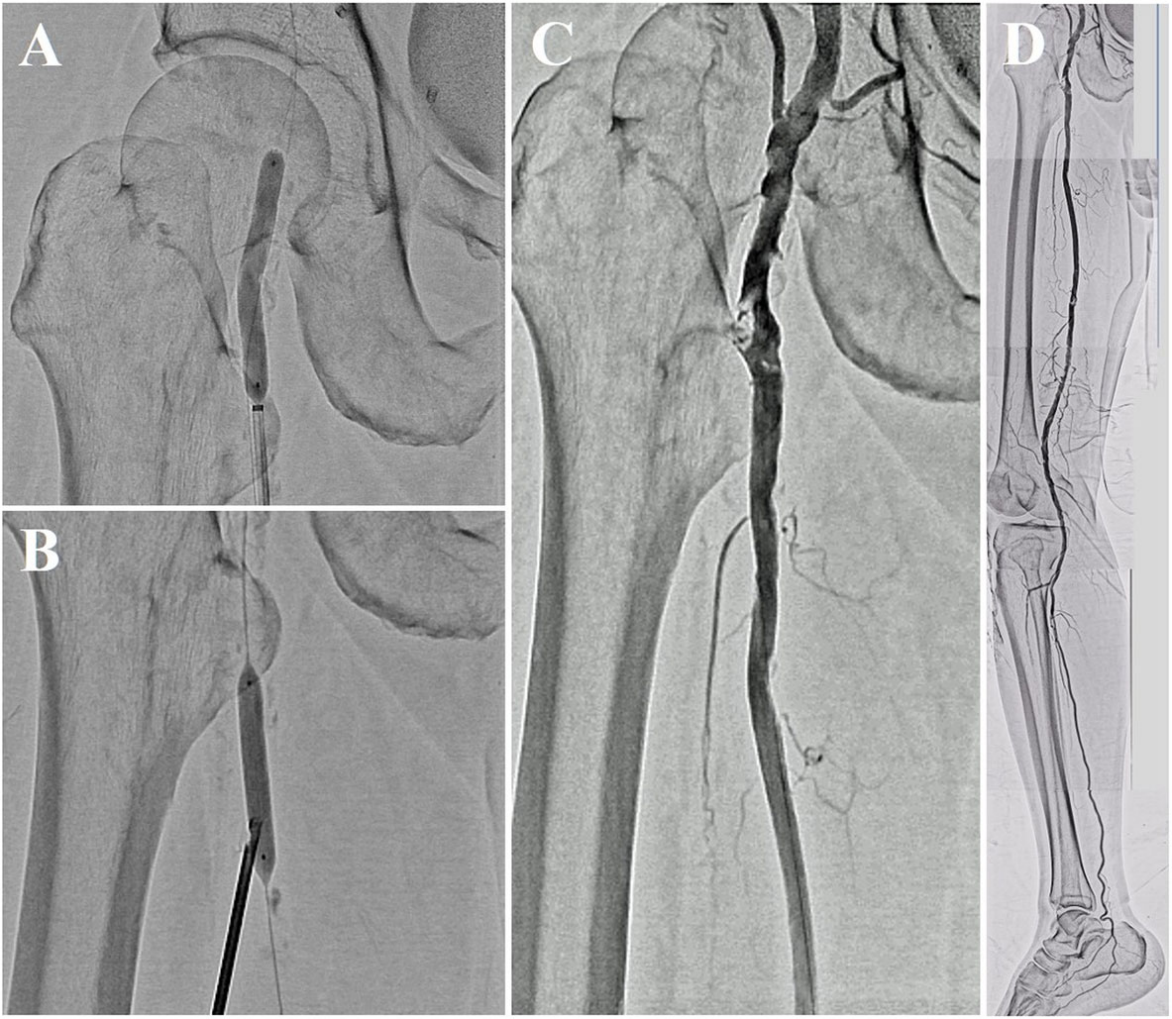
**A** Inflation of a 6-mm drug-coated balloon catheter in the common femoral artery (CFA). **B** Hemostasis of the puncture site of the superficial femoral artery with vascular suture assisted by balloon inflation inside the vessel. Final angiography image demonstrating a reduced filling defect of contrast medium in the CFA (**C**) and favorable blood flow as far as below the ankle (**D**)

**Fig. 4:**
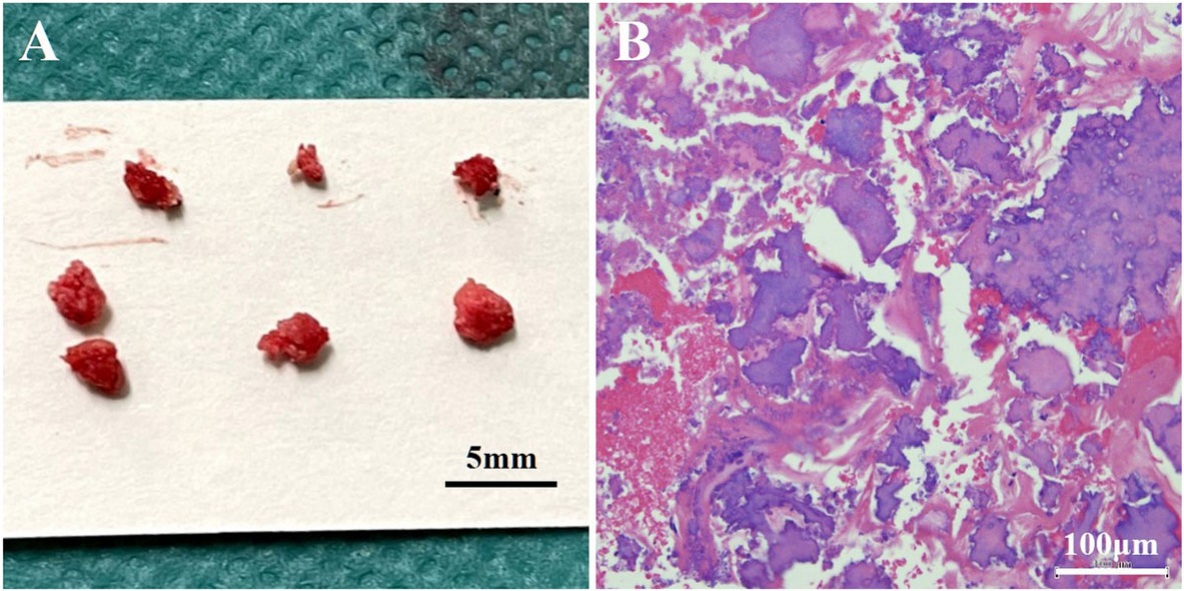
**A** The collected specimens of various sizes and rigidities. **B** Histopathology demonstrating a calcified plaque mixed with fibrin deposition to various extents

Finally, we inflated a 6-mm drug-coated balloon catheter after inflating a 6-mm scoring balloon catheter (Fig. 3 A). The SFA puncture site was closed with a Perclose ProGlide Suture-Mediated Closure System (Abbott Laboratories, Lake Bluff, IL, USA) assisted by balloon inflation (Senri; Terumo Corp., Tokyo, Japan) inside the vessel for 3 min (Fig. 3B), which allowed the patient to be ambulatory immediately after the procedure without requiring bed rest. The final angiography demonstrated a reduced filling defect of contrast medium in the CFA (Fig. 3 C) and favorable blood flow below the ankle (Fig. 3D). The collected specimens had various sizes and rigidities, and the maximum sample diameter was approximately 3.2 mm (Fig. 4 A). Histopathology with hematoxylin and eosin staining showed calcified plaques mixed with fibrin deposition to various extents (Fig. 4B). After treatment, the right ABI recovered to 0.89 and the claudication resolved completely (Rutherford class 0). After discharge, the patient continued the same prescriptions. One month later, the right ABI remained at 0.95, and no symptoms of claudication were observed (Rutherford class 0).

## Discussion

Here we demonstrated a less invasive and successful EVT procedure for severe nodular calcification in CFA. Atherectomy devices are adjunctively important for debulking heavily calcified plaques (Panaich et al. 2016), and a greater plaque volume reduction usually results in more efficient balloon angioplasty and/or stenting (Böhme et al. 2021; Hassan et al. 2021). Various types of debulking devices are now available in most countries, but unfortunately, none designed for EVT are covered by insurance in our country. Therefore, we devised this original method to obtain favorable vessel dilation for severe nodular calcifications in the CFA as an alternative to conventional atherectomy devices.

Our strategy has three strengths. First, the use of myocardial biopsy forceps enabled repetitive and effective debulking despite the presence of a hard calcified mass. However, vessel rupture and distal embolism are major complications requiring prevention. In this regard, owing to the closer position between the balloon-guiding catheter and the targeted plaque, we easily delivered the forceps just distal to the target, intentionally targeting the plaque and optimizing their use. To ensure safe debulking, clinicians should occasionally assess the lesion using intravascular imaging modalities and determine the maneuver’s endpoint. Of course, we must acknowledge that our method’s use of biopsy forceps is technically off-label.

Second, the OPTIMO PPI balloon-guiding catheter completely prevented distal embolization. As this balloon-guiding catheter was developed to prevent thromboembolism, applying the system in this case was reasonable. However, we must consider the risk of balloon rupture of this catheter during procedure because continuous friction occurs between the balloon and vessel wall due to the repetitive debulking maneuvers.

Third, avoiding puncturing the bilateral CFA allowed the patient to be ambulatory immediately after the procedure without requiring bed rest. Severe calcification of the CFA often occurs bilaterally, and the contralateral femoral approach was not feasible in our case.

In such circumstances, we can use the R2P system (Terumo Corp., Tokyo, Japan), an EVT device specialized for the trans-radial approach (Shinozaki et al. 2019).

We emphasize that this strategy is very patient-oriented since it leaves nothing behind, the patient does not require postoperative bed rest, and maximum debulking of nodular calcification is achieved. Only one other case report to date described the use of myocardial biopsy forceps for debulking nodular calcification in CFA (Tanaka et al. 2020). However, our method is distinct from that of the previous report since it combines use of the R2P system and the OPTIMO catheter in the proximal SFA, which can contribute to patient comfort and more efficient debulking. We named this procedure the CRUNCH (CRUsh Nodular Calcification with easy Hemostasis management) technique.

## Conclusions

A severely calcified lesion in the CFA is usually difficult to treat using an endovascular strategy, but our novel less invasive and effective debulking strategy using myocardial biopsy forceps -called the CRUNCH technique- may become a promising technique for such situations.

## Supplementary information

**Additional file 1: Video 1.** Debulking nodular calcification performed with forceps under distal protection using a balloon-guiding catheter

**Additional file 2: Video 2.** Difficulty detaching a calcified hard mass on a large scale using this procedure

**Additional file 3**

## Data Availability

Not applicable.

## Abbreviations

ABI: Ankle-brachial pressure index
CFA: Common femoral artery
CFE: Common femoral endarterectomy
EVT: Endovascular treatment
IVUS: Intravascular ultrasonography
PAD: Peripheral artery disease
RA: Radial artery
SFA: Superficial femoral artery
