# Supplementary material for: Percutaneous debulking strategy for severe nodular calcification in common femoral artery
Source: CVIR Endovasc. 2022 May 27;5:25. doi: 10.1186/s42155-022-00301-6 (PMC9142719; doi:10.1186/s42155-022-00301-6)
Supplement: Supplementary file 3 — Additional file 3 [file 42155_2022_301_MOESM3_ESM.docx]

**May 3. 2022.**

**Title: Percutaneous Debulking Strategy for Severe Nodular Calcification in Common Femoral Artery**

**Ms. Ref. No.: CIRE-D-22-00040R1**

Dear Editor,

Thank you for consideration of our manuscript for publication in your journal.

We have revised our manuscript according to your sincere comments.

Number of the pages and lines is of the pre-revised manuscript.

*Reviewer #1:*

*This is an interesting report. I suggest some modifications in order to improve the readability. Moreover, some pieces of information about the case may be added.*

*General comments*

*1. The manuscript can be shortened in some points. Mostly, the discussion can be cut. You can focus on the innovative aspects of your research without discussing aspects/techniques which are well known by the readership of CVIR Endovascular.*

→ According to your advice, we cut some sentences and made the description more simple mainly in the “Discussion” section.

*2. I suggest to specify that the usage of the forceps was off-label*

→　We added the description about the information of biopsy forceps regarding off-label use in Page 7 Line 19.

*3. The quality of language needs to be improved. I suggest to ask to some more experienced colleagues or to use a language editing services.*

→　We had our manuscript professionally edited by commercial service. (editage: <https://www.editage.com/>) and added the description in the Acknowledgement.

*Specific comments*

*Abstract*

*P2-L19 The word "massive" is not necessary and too colloquial. It is used several times to describe your technique. I would also mention the aspiration of the material, as part of your technique, and the positive follow-up data.*

→　According to your suggestion, we changed the word “massive” to “effective” throughout the manuscript. In this paper, we want to show the effectiveness of this technique as a debulking device for severe nodular calcification in the CFA.

*P3-L8 "Less invasive". Do you mean less invasive than surgery? Comparing with other endovascular techniques, your method can be considered quite invasive.*

→　Thank you for your comments. In this manuscript, we consider that our method is less invasive than the typical treatment option “endarterectomy” for the CFA nodular calcification. The reason why we consider it less invasive than the surgery is that our method can be carried out under local anesthesia, it usually takes less time in EVT procedure than the surgery, the patient is ambulatory immediately after the procedure without requiring bed rest, and the patient can discharge on the next day of the procedure. Moreover, some drawbacks of common femoral endarterectomy, such as wound complications, have been reported (Nguyen et al. 2015). We added this citation in the “Introduction” section in Page 4 Line 19.

*Introduction*

*P4-L11/19 This is practically the same background of the abstract. I suggest to rephrase it.*

→　Thank you for your suggestion. We entirely rephrased the contents in the “Introduction” section.

*Case presentation*

*Can you provide the medication history of the patient before the intervention and the medication history after the discharge?*

→　We added the information about medication history in Page 4 Line 31 and Page 6 Line 11.

*Have you performed some other imaging other than ultrasound before the intervention?*

→　The patient underwent coronary intervention via radial artery a few months before this EVT. At that time, we conducted angiography of lower extremities and evaluated the lesion. So, we have not performed any other imaging other than ultrasonography in advance.

*Did you administer Heparin during the intervention?*

→　We administered intra-arterial bolus of 5,000 units of unfractionated heparin and adjusted the activated clotting time to > 250 s during the procedure. We added the description about it in Page 4 Line 56.

*P4-L39 Please specify the acronym CFE*

→　We spelled out the word “common femoral endarterectomy” in the “Introduction” section.

*P4-L49 I guess it is right instead of left.*

→　We surely approached via left radial artery. We usually adopt this manner in using R2P system because the distance from the left side is shorter than the right side as far as lower extremity and it is sometimes difficult to pass over the aortic arch from the right side.

*P5-L45 "only". The usage of this word is not clear*

→　We deleted the word according to your advice.

*Discussion*

*P6-L39/45 I suggest a linguistic revision of this sentence.*

→ According to your suggestion, we revised the sentence.

*P7-L14/19 I suggest a linguistic revision of this sentence.*

→ According to your suggestion, we revised the sentence.

*The paragraph from P6-L47 to P7 L20 is quite verbose and can be shortened.*

*The paragraph P7-L44 to P8-L8 can be cut/shortened because it regards aspects which are commonly known.*

→ According to your advice, we cut some sentences and made the description more simple.

*Images and videos*

*No suggestions. Everything well documented. Nice job.*

→ Thank you for your compliments.

**May 3. 2022.**

**Title: Percutaneous Debulking Strategy for Severe Nodular Calcification in Common Femoral Artery**

**Ms. Ref. No.: CIRE-D-22-00040R1**

Dear Editor,

Thank you for consideration of our manuscript for publication in your journal.

We have revised our manuscript according to your sincere comments.

Number of the pages and lines is of the pre-revised manuscript.

*Reviewer #2:*

*Thank you for allowing me to review your manuscript. My comments can be seen below:*

*1) Figure 1: Please crop part B of the figure again. The image in part D is not clear-- please choose a better image if possible.*

→　I appreciate your advice. I cropped the part B of Figure 1. The image in part D is really unclear as you mentioned. However, this image is the best one of all, so we are sorry we cannot replace it.

*2) Abstract Conclusions: I would remove the statement: depending on the conditions. and say may become a promising technique for the management of these lesions in the future.*

→　Thank you for your kind suggestion. We have adopted your opinion.

*3) The introduction of the abstract and the paper is the same. I would recommend some modification and adding a bit more background in the full manuscript introduction.*

→　Thank you for your suggestion. We entirely rephrased the contents in the “Introduction” section.

*4) Case presentation: Page 4, line 30: Please add Rutherford classification*

→　We added the information of Rutherford classification at the relevant part.

*5) Case presentation: Page 4, line 39: Expand CFE for the first time*

→　We spelled out the word “common femoral endarterectomy” in the “Introduction” section.

*6) Case presentation: Page 4, line 45: I would recommend adding a better justification for why EVT was performed for this patient rather than just saying they refused to undergo surgery.*

→　According to your advice, we revised the description about the reason for patient’s treatment option.

*7) Case presentation: Page 4, line 58: Keep the company, location description consistent- in this line the location of terumo is not mentioned*

→　We added the company information of Terumo in Page 4 Line 58, Page 5 Line 42, and Page 8 Line 5.

*8) Case presentation: Page 5, line 5: mention ipsilateral SFA was used for second access?*

→　We added the explanation of the proximal SFA puncture as the ipsilateral retrograde access.

*9) Case presentation: Page 5, line 14: what is NC? Please expand*

→　We spelled out the word “nodular calcification” throughout the manuscript.

*10) Case presentation: Page 5, line 22: Please mention which distal protection device was used*

→　As we described in the sentence, we used OPTIMO PPI balloon-guiding catheter as a distal protection device.

*11) Case presentation: Page 6, line 10: Add Rutherford classification post procedure and on follow-up*

→　We added the information of Rutherford classification at the relevant part.

*12) Case presentation: Page 6, line 22: How is this less invasive? if the NC was "crunched" 43 times?*

→　Thank you for your comments. In this manuscript, we consider that our method is less invasive than the typical treatment option “endarterectomy” for the CFA nodular calcification. The reason why we consider it less invasive than the surgery is that our method can be carried out under local anesthesia, it usually takes less time in EVT procedure than the surgery, the patient is ambulatory immediately after the procedure without requiring bed rest, and the patient can discharge on the next day of the procedure. Moreover, some drawbacks of common femoral endarterectomy, such as wound complications, have been reported (Nguyen et al. 2015). We added this citation in the “Introduction” section in Page 4 Line 19.

*13) Case presentation: Page 7, line 46: Add citation on improved ambulation if bilateral CFA are not punctured*

→　In this sentence, we just want to explain the fact that the patient could walk immediately after the procedure without requiring bed rest due to avoidance of puncture in the bilateral CFA. We do not intend to express the improved ability of ambulation.

*14) Case presentation: Page 8, line 11: Wasn't some plaque still left behind?*

→　In the field of endovascular treatment, “leave nothing behind” means the interventional strategy that we finalize the procedure without deploying any stents. Drug-coated balloon dilation is usually adopted in most cases. In our case, we completed our procedure with “leave nothing behind” strategy.
